# Apical Hypertrophic Cardiomyopathy With a Sub-aortic Membrane: A Case Report

**DOI:** 10.7759/cureus.52846

**Published:** 2024-01-24

**Authors:** Akbar Hussain, Adnan Bhopalwala, Huzefa Bhopalwala, Nakeya Dewaswala, Aelia Akbar

**Affiliations:** 1 Internal Medicine, Appalachian Regional Health, Harlan, USA; 2 Internal Medicine, Appalachian Regional Healthcare, Whitesburg, USA; 3 Cardiovascular, Mayo Clinic, Rochester, USA; 4 Cardiovascular Medicine, University of Kentucky, Lexington, USA; 5 Public Health, Loyola University Medical Center, Chicago, USA

**Keywords:** heart surgery, hypertrophy cardiomyopathy, subaortic stenosis, apical hypertrophy cardiomyopathy, hypertrophic obstructive cardiomyopathy (hocm)

## Abstract

Hypertrophic obstructive cardiomyopathy (HOCM) and subaortic membrane (SAS) are distinct cardiac conditions, but their coexistence presents complex diagnostic challenges. We report the case of a 52-year-old male with HOCM and a concurrent subaortic membrane, highlighting the intricacies of diagnosis and management. The patient's presentation included symptoms of dyspnea and chest tightness, and diagnostic evaluations revealed a unique combination of dynamic left ventricular outflow tract (LVOT) obstruction from HOCM and fixed obstruction from the subaortic membrane. This case emphasizes the importance of a comprehensive diagnostic workup to guide appropriate treatment decisions when managing multiple cardiac abnormalities.

## Introduction

Hypertrophic obstructive cardiomyopathy (HOCM), historically known as idiopathic hypertrophic subaortic stenosis, is a relatively common genetic disorder with a high risk of sudden cardiac death, impacting young individuals, including athletes, of all races and genders. It's a common, heritable cardiovascular disease affecting approximately 1 in 500 individuals [1]. It primarily stems from asymmetric septal hypertrophy in the left ventricle, often obstructing blood flow. HOCM is challenging to diagnose and is frequently detected only after a severe cardiac event. It can affect any left ventricular segment but typically manifests in the interventricular septum, and it usually remains asymptomatic in children until potentially fatal complications arise in teenagers and adolescents [2]. This condition becomes even more challenging to manage clinically when a subaortic membrane is present. Subaortic stenosis (SAS) is a condition where there is an obstruction of the left ventricular outflow tract (LVOT). It can be categorized into two types: discrete membranous SAS and diffuse fibromuscular SAS [3]. While hypertrophic cardiomyopathy (HCM) and SAS are typically seen as separate conditions, there are rare cases where they coexist, posing a diagnostic challenge for healthcare providers [4]. In this case report, we present the comprehensive cardiometabolic evaluation and management of a middle-aged male patient who presented with HOCM and a concomitant subaortic membrane.

## Case presentation

We present the case of a 52-year-old male patient with a history of hypertension, hyperlipidemia, and a remote history of underground coal mining who presented with symptoms of dyspnea and chest tightness for five days. He had been diagnosed with HOCM and had started beta-blocker therapy with significant symptom improvement.

On physical examination, the patient presented with no acute distress. Vital signs were within normal limits, with stable blood pressure and heart rate. Cardiac auscultation revealed a regular rhythm with no murmurs, and lung auscultation was unremarkable. Examination of the chest revealed no signs of chest wall deformities or masses. The abdomen was soft and non-tender, with no hepatomegaly or splenomegaly detected. Peripheral pulses were palpable and symmetric. Neurological examination demonstrated intact cranial nerves and no focal deficits. Notably, there were no signs of lower extremity edema or jugular venous distension. Overall, the physical examination findings were consistent with a stable cardiovascular status and did not reveal any acute concerns.

On further evaluation, the Holter monitor showed intermittent atrial tachycardia, which prompted further investigation. Echocardiogram findings included asymmetrical septal hypertrophy, diastolic filling within the normal range, and suspected systolic anterior motion of the anterior mitral leaflet. Other tests revealed mild aortic valve regurgitation and moderate to severe aortic valve stenosis, fixed LVOT obstruction caused by a subaortic membrane, and dynamic LVOT obstruction due to systolic anterior motion (SAM) of the mitral valve-a hallmark feature of hypertrophic cardiomyopathy (HCM). Additionally, the echocardiogram showed a trileaflet aortic valve with restricted opening, moderate aortic regurgitation, and mild mitral regurgitation. The patient was awaiting a cardiac MRI for further risk assessment. Imaging studies showed findings consistent with his diagnosis.

**Figure 1:**
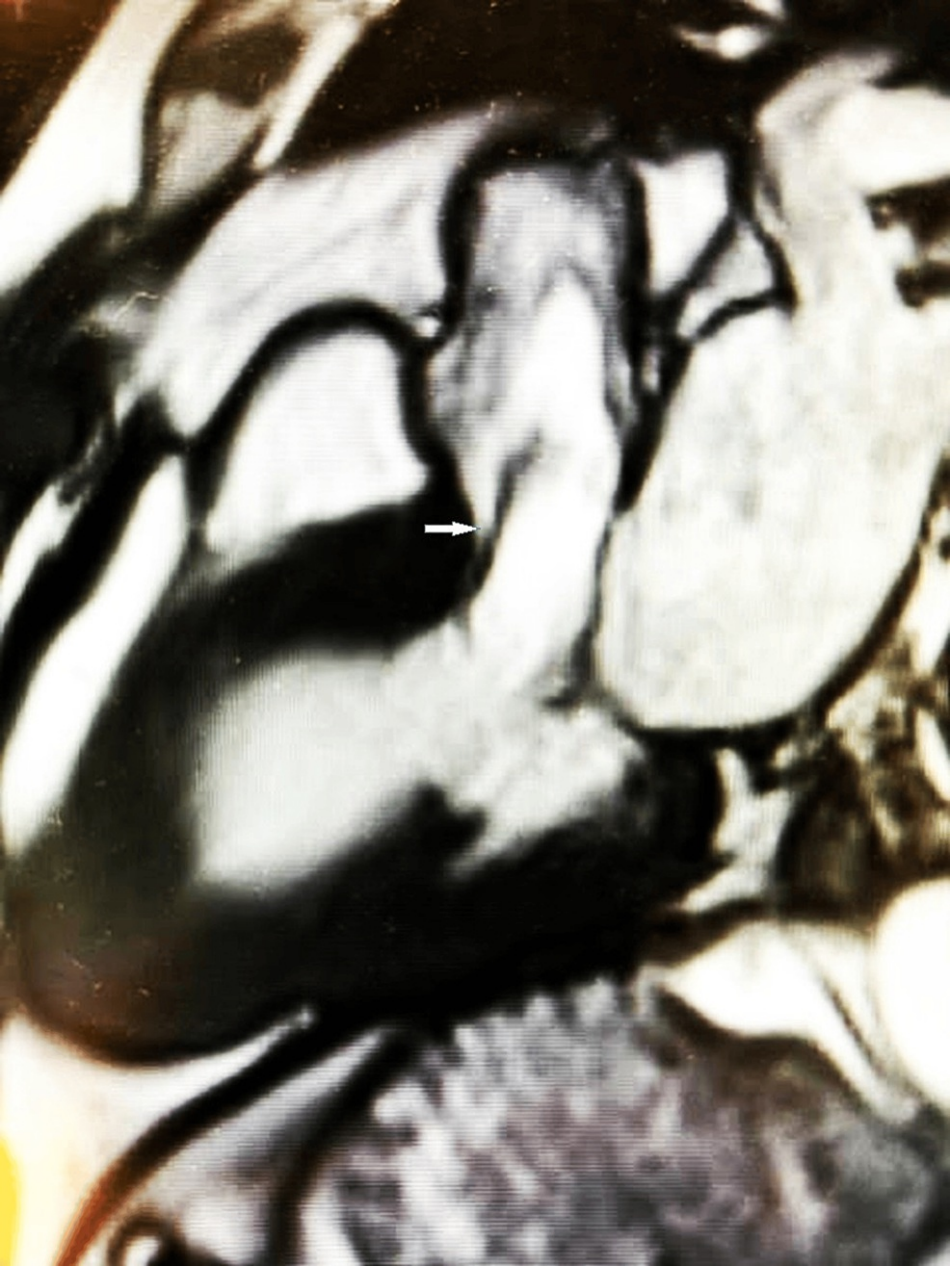
Cardiac MRI showing a sub-aortic membrane (white arrow)

The cardiac MRI findings revealed the patient's diagnosis of apical HCM and the presence of a sub-aortic membrane as shown in Figure 1. The MRI showed no late gadolinium enhancement (LGE), indicating a lack of myocardial scarring or fibrosis. The left ventricle (LV) and right ventricle (RV) were both normal in size with preserved systolic function. Specifically, the LV ejection fraction (EF) was 67%, and the RV EF was 54%. These findings confirmed the apical HCM diagnosis. Additionally, a sub-aortic membrane was identified, causing flow acceleration in the left ventricular outflow tract (LVOT) with a peak velocity of 2.1 m/s. This comprehensive cardiac MRI provided valuable insights into the patient's cardiac structure and function, contributing to the accurate diagnosis and management of their condition.

The personalized treatment plan included, in response to the patient's improved symptoms following the initiation of metoprolol, a comprehensive management plan. The first step involved discontinuing lisinopril-hydrochlorothiazide to streamline the patient's medication regimen. Due to the presence of a sub-aortic membrane and the potential risk of exacerbating symptoms, the patient was advised to avoid dehydration and the use of diuretics. Metoprolol tartrate, which had shown efficacy in symptom management and resulted in a resting heart rate in the 50s, was to be continued at the maximum tolerated dose. Additionally, verapamil was introduced at a daily dosage of 180 mg to further address the dynamic LVOT obstruction seen in the patient. The long-term follow-up plan included an annual resting transthoracic echocardiography (TTE) to monitor cardiac function and structure, as well as the suggestion of an exercise stress echocardiogram to assess gradients during exertion. Annual Holter monitoring would track any arrhythmias. Importantly, it was emphasized that surgical intervention for the sub-aortic membrane would not be pursued unless significant aortic regurgitation, left ventricular systolic dysfunction or a resting gradient exceeding 50 mmHg was detected, thus providing a tailored and patient-centered approach to managing this complex cardiovascular condition.

## Discussion

HCM, characterized by myocardial wall thickness exceeding 15 mm in one or more left ventricular segments, is a relatively common genetic cardiac disorder with autosomal dominant inheritance, impacting approximately one in 500 individuals in the general population [5,6]. In contrast, subaortic membranes are rare congenital heart abnormalities, typically composed of thin fibrous or occasionally muscular tissue in the LVOT [7]. The coexistence of a subaortic membrane with HCM is rare, reported in about 1.7% of cases with LVOT obstruction, making it a challenging diagnosis [8].

While both HCM and subaortic membrane-related LVOT obstruction can lead to left ventricular hypertrophy, they can be differentiated by key characteristics. HCM is characterized by asymmetric septal hypertrophy as a primary pathological change, whereas the subaortic membrane primarily causes left ventricular concentric hypertrophy as a secondary pathological change. Additionally, systolic anterior motion (SAM) of the mitral valve, a diagnostic feature of HCM, is not observed in subaortic membrane-related LVOT obstruction.

The patient's echocardiogram in this case revealed a coexistent dynamic LVOT obstruction from HCM and a fixed obstruction from a subaortic membrane. Identifying this dual pathology demands a high index of suspicion and has significant implications for management and prognosis. Patients with both HCM and subaortic membranes may face an increased risk of progressive heart failure symptoms [4]. Accurate diagnosis is crucial because treatment options and prognostic considerations differ substantially, including the risk of sudden death and the need for family screening [7].

Echocardiography is a pivotal diagnostic tool for assessing subaortic membranes, typically presenting as a strong linear echo in the LVOT below the aorta or a hypertrophic muscular structure protruding into the LVOT [3]. Specific echocardiographic views, such as the parasternal long axis (PLAX), apical long axis (APLAX), and apical five-chamber (AP5CH), are essential for diagnosing SAM of the mitral valve, a hallmark of HCM. Additionally, cardiac MRI and genetic testing can contribute to HCM diagnosis while ECG findings, including precordial voltages and non-specific ST segment and T-wave abnormalities, can aid in identifying HCM.

In this case, the patient's echocardiogram demonstrated severe asymmetric left ventricular hypertrophy, SAM of the mitral valve, and aortic valve dysfunction due to a subvalvular membrane. Accurate timing of ECG with the echocardiogram is critical for identifying SAM. Distinguishing the underlying cause of heart failure is essential, as it influences treatment approaches and prognostic considerations. HCM is typically treated with surgical septal resection or percutaneous alcohol ablation while subaortic membranes necessitate surgical removal. Conservative treatments for HCM may include beta-blockers while HCM combined with SAS may require low-dose diuretics for symptom management, given the more severe LVOT obstruction [2].

## Conclusions

Conclusion:

The presented case of a 52-year-old male with concurrent apical hypertrophic cardiomyopathy (HCM) and a subaortic membrane (SAS) exemplifies the diagnostic complexities and nuanced management strategies required when dealing with rare cardiac comorbidities. The successful diagnosis hinged on an array of advanced cardiac imaging modalities, genetic testing, and electrocardiography, underlining the significance of a comprehensive diagnostic approach. The management plan, tailored to the patient's specific clinical presentation, highlighted the importance of individualized care, incorporating medications like beta-blockers and verapamil to address HCM-related symptoms while judiciously reserving surgical intervention for the subaortic membrane based on well-defined criteria. This case underscores the crucial role of interdisciplinary collaboration among healthcare specialists and the need for ongoing patient monitoring to adapt treatment strategies as necessary.
